# Metabolic Bone Disease in Viral Cirrhosis: A Prospective Study

**DOI:** 10.1155/2013/276563

**Published:** 2013-04-21

**Authors:** Rabia Goubraim, Nawal Kabbaj, Mouna Salihoun, Zakia Chaoui, M'Hamed Nya, Naima Amrani

**Affiliations:** EFD-Hepatogastroenterology Unit, Ibn Sina University Hospital, Rabat 10000, Morocco

## Abstract

*Background/Aim*. Metabolic Bone disorders are well-recognized extrahepatic complications of cirrhosis. The aim was to report their prevalence and the associated factors to their development in patients with viral cirrhosis. *Patients and Methods*. All consecutive patients with viral cirrhosis were prospectively enrolled. Parathyroid hormone, 25-hydroxyvitamin D, liver function, and phosphocalcic tests were measured in all patients. Bone mineral density was measured at the lumbar spine and total hip by dual-energy X-ray absorptiometry. Data were analyzed using SPSS software. *Results*. Forty-six cirrhotic patients were included with hepatitis C (87%) and hepatitis B (13%). The Child-Pugh score was grade A in 87% of cases and grade B in 13%. Thirty-seven patients had decreased bone mineral density with osteopenia in 24 patients and osteoporosis in 13 patients. Decreased 25-hydroxyvitamin D was found in 95.6% of cases. Bone disorders were significantly more frequent in old patients with low body mass index, long duration of liver disease, and low 25-hydroxyvitamin D level. None of these factors was an independent factor associated with bone disorders. *Conclusion*. Our study revealed a high prevalence of metabolic bone disorders among viral cirrhotic patients. Consequently, bone mineral density assessment should be performed systematically in all cirrhotic patients.

## 1. Introduction

The association between metabolic bone abnormalities and chronic liver disease is now well recognized. Often known as hepatic osteodystrophy, these disorders are a common complication of liver cirrhosis with a reported prevalence of 12% to 86% [1–4]. Osteoporosis is the well-known major complication of hepatic osteodystrophy. Its prevalence varies considerably and ranges from 20% to 50% [5]. It is related to the severity of cirrhosis and affects 38% of patients awaiting liver transplantation [6, 7]. Osteoporosis is characterized by low bone mass and microarchitectural deterioration of bone tissue leading to bone fragility and increased fracture risk, a source of morbidity and mortality in patients already weakened by their chronic liver disease.

The pathogenesis of bone demineralization in cirrhosis remains incompletely understood. It is most often multifactorial and many factors affecting directly or indirectly bone turnover have been implicated: insulin growth factor-I, interleukin-1, tumor necrosis factor-*α*, and osteoprotegerin, which promote osteoclastic bone resorption in addition to the receptor activator of NF kappa beta (RANK) and the receptor activator of NF kappa beta ligand (RANKL).

The serum level of these factors is disturbed during cirrhosis leading to a decrease in osteoblast activity and increased bone resorption by osteoclasts, which is responsible for a decrease in bone mineral density in these patients.

These disorders have been studied mainly in cholestatic liver diseases such as primary biliary cirrhosis and less frequently in viral hepatitis.

The aim of this prospective study was to report the prevalence of metabolic bone disorders (MBDs) in a cohort of Moroccan patients with viral B and C cirrhosis, to study their characteristics and to identify the associated factors to their development. 

## 2. Patients and Methods

### 2.1. Patients

Between January 2011 and May 2012, all consecutive patients with viral B and C cirrhosis regularly followed in our unit were enrolled, in a prospective, monocentric, descriptive, and analytical study.

All included patients fulfilled the following criteria:

- age over or equal to 18 years;
- liver cirrhosis proven histologically, or diagnosed using noninvasive markers of fibrosis (FibroTest or Fibroscan) or diagnosed on clinical, biological (hypoalbuminemia, reduced prothrombin activity), echographic (dysmorphic liver, portal hypertension's signs, ascites), and endoscopic (oesophageal or gastric varices, portal hypertensive gastropathy) criteria;
- B and C viral etiology of cirrhosis, respectively, defined by the presence of serum hepatitis B surface Antigen (HbsAg) or hepatitis C virus antibodies.

Patients with other etiology of liver cirrhosis, those coinfected by human immunodeficiency virus or presenting endocrine disorders or renal failure, and patients receiving medications that could influence bone metabolism (bisphosphonates, estrogens, androgens, corticosteroids, vitamin D, or calcium supplementation) were excluded from the study.

### 2.2. Clinical Aspects and Liver Status

The following clinical items were collected at enrolment for each patient: age, gender, height, weight, body mass index (BMI), and the duration of the liver disease. All patients underwent liver function tests including serum bilirubin, alanine, and aspartate aminotransferase activities, alkaline phosphatase, gamma-glutamyl transferase in addition to prothrombin time, and serum albumin using standard laboratory methods. The severity of cirrhosis was evaluated by the Child-Pugh score that allows the classification of patients into three grades of increasing severity: A (5-6), B (7–9), and C (10–15).

### 2.3. Biochemical Bone Metabolism Tests

Serum calcium, serum phosphorus, 24 hours urinary calcium, and phosphorus were measured in all patients with an enzymatic colorimetric assay method.

25-Hydroxyvitamin D (25 (OH) D) was measured using chemiluminescence immunoassay, normal ranges were between 20 and 70 *μ*g/L, insufficiency was defined as 25 (OH) D level below 20 *μ*g/L and deficiency as below 10 *μ*g/L. Intact parathyroid hormone 1–84 (iPTH) was measured with chemiluminescence immunometric method, and normal ranges were between 15 and 65 pg/mL.

### 2.4. Bone Mineral Density Measurements

Bone mineral density (BMD) was measured in all patients at the lumbar spine (L1–L4) and total hip using dual energy X-ray absorptiometry DXA (Lunar Prodigy, General Electric Healthcare, USA). Results were expressed in *T*-score (difference in standard deviation (SD) between the patient's measured BMD value and the maximum mean BMD of young adult of same gender) and *Z*-score (difference in SD between the patient's measured BMD value and the normal reference for age and gender). According to the World Health Organization criteria (WHO) [8], osteoporosis was defined as a *T*-score below −2.5 SD and osteopenia as *T*-score between −1 and −2.5 SD.

### 2.5. Statistical Analysis

Continuous variables were expressed as mean ± SD if they are normally distributed, and if not they were expressed as median (quartiles). Categorical variables were expressed as numbers and percentages. Pearson's chi-square test (*χ* ^2^) and Fisher's exact test for categorical variables, Student's test for normally distributed continuous variables, and Mann-Whitney test for nonnormally distributed continuous variables were applied to analyze differences between patients. Data analysis was performed using SPSS software version 18.0 for Windows (SPSS Inc., Chicago, II). A two-tailed *P* value <0.05 was considered statistically significant.

## 3. Results

Forty-six patients with viral cirrhosis were included in the study. There were 26 men (56%) and 20 women (44%). The mean age of patients was 64 ± 9.16 years (range: 36–82 years). Forty patients (87%) had hepatitis C related cirrhosis and 6 patients (13%) had postviral hepatitis B cirrhosis. The mean duration of the liver disease was 9.9 ± 5.37 years. Forty patients (87%) were classified grade A of Child-Pugh classification and 6 patients (13%) were grade B. The mean BMI was 23.25 ± 2.97 Kg/m². Biological data of our patients are summarized in Table 1. The mean level of serum 25 (OH) D was 13.2 ± 5.1 *μ*g/L (range: 4–25.4 *μ*g/L). Decreased 25 (OH) D was found in 44 patients (95.6%), and 15 of them (32.6%) had vitamin D deficiency and 29 patients (63%) had vitamin D insufficiency. Two patients (4.4%) had normal levels of vitamin D. Among patients with decreased vitamin D, 13 (28.2%) had secondary hyperparathyroidism with PTH levels between 70 pg/mL and 217 pg/mL.

Thirty-seven patients (80.4%) out of 46 had decreased BMD, and 24 (52.2%) of them had osteopenia, while 13 (28.2%) had osteoporosis at least at one site. Nine patients (19.6%) had normal BMD.

Osteopenic patients (*n* = 24) included 10 women and 14 men (mean age: 64.2 ± 9.3 years) infected with hepatitis C virus in 21 cases and hepatitis B virus in 3 cases. Osteoporotic patients (*n* = 13) included 7 women and 6 men (mean age: 67.8 ± 6 years) with viral hepatitis C in 12 cases and hepatitis B in one case.

BMD varied depending on the measurement site. Bone loss was more frequent and more severe in the lumbar spine. At the lumbar spine, 31 patients (67.4%) had decreased BMD with osteopenia in 21 cases (45.7%) and osteoporosis in 10 cases (32.7%).

At the total hip, bone loss was found in 27 patients (58.7%), and 20 (43.5%) of them had osteopenia and 7 (15.2%) had osteoporosis. BMD's results expressed in g/cm^2^, *T*-score, and *Z*-score are summarized in Table 2.

*T*-scores were significantly different between men and women at the lumbar spine (*P* = 0.02) and total hip (*P* = 0.04); accordingly, BMD was significantly lower in women in both sites with *P* = 0.007 in the lumbar spine and *P* = 0.01 in total hip (Table 3).

Patients were divided into 2 groups: low BMD (osteopenia/osteoporosis) group and normal BMD group and were compared (Table 4). Patients with low BMD were significantly older than those with normal BMD (*P* = 0.02), and they had a lower BMI (*P* = 0.01), a long duration of liver disease (*P* = 0.04), and their vitamin D level was lower (*P* = 0.01).

In univariate analysis, age, BMI, duration of liver disease, and vitamin D level were associated with the presence of osteoporosis and osteopenia. In multivariate analysis, none of these variables was an independent factor associated with metabolic bone disorders in cirrhotic patients (Table 5).

During the study period, no pathological fracture has occurred in our patients. In case of bone demineralization assessed by DXA, a systematic treatment was given to patients. Calcium and vitamin D supplementations were prescribed in case of osteopenia. In addition, bisphosphonates were prescribed in cases of established osteoporosis.

## 4. Discussion

With progress in the therapy of liver cirrhosis and its complications, there has been an increase in patient survival and, hence, an increased incidence of metabolic bone disorders especially osteoporosis and its fracture risk. The prevalence of these osteometabolic changes in chronic liver disease varies from 13% to over 80%, depending on the population studied and the diagnostic criteria used to define bone disease [4, 9, 10].

This prospective study assessed BMD, measured using the reference radiological method, and defined according to the WHO's criteria. It showed a high prevalence of BMD abnormalities in the evaluated cirrhotic patients with a rate of 80.4%. Osteoporosis was found in 28.2% of cases and osteopenia in 52.2% of cases with a higher prevalence in the lumbar spine.

Our results were similar to those found in a previous study [11] who noted low BMD in 80% of a comparable patient's population. Two Egyptian studies recently published have also found high rates of bone loss in patients with cirrhosis: 86.6% in Ahmed et al.'s study [4] and 87.8% in Salama et al.'s series [12]. These findings allow us to suggest that Moroccan cirrhotic patients have approximately the same osteodensitometric profile to the rest of North-African population.

In Javed et al.'s study [13] which included 100 Pakistani patients with viral hepatitis related cirrhosis, the prevalence of metabolic bone disease was 68%. A similar figure has been reported by the authors of an Indian study [10]. This rate was lower than that found in our study, and this is probably due to the ethnic difference between the populations studied, but the prevalence of osteoporosis observed in our patients was similar to the rate reported by Javed et al. (28.3% versus 26%). Other series have found much higher osteoporosis prevalence. Gonzalez-Calvin et al.'s series [14] which showed osteoporosis in 56% of cases and the study of Salama et al. [12] reported an osteoporosis rate of 43.9%. This is probably due to the very good liver function of our patients in contrast of the two other studies. Indeed, the level of liver failure is correlated with the risk of osteoporosis, and the progression of liver disease from grade A to grade C of the Child-Pugh classification is associated with an increase in bone loss [15].

We noticed like other series [10, 14, 16] that bone loss in lumbar spine (trabecular bone) was more frequent and more severe than in the hip (cortical bone). This finding can be explained by a much lower turnover of cortical bone compared to trabecular bone [17], and probably some risk factors for osteoporosis have lesser deleterious effects on bone with lower rates of turnover.

The mechanism of bone mass loss in viral cirrhosis is not fully understood; it is probably multifactorial, and several risk factors may be involved. Tumor necrosis factor-alpha [14] and transforming growth factor-*β* [18] have been implicated in the bone injury during viral hepatitis.

Insulin-like growth factor-I which is produced by the liver and the bone and stimulates osteoblast's proliferation was found decreased in viral cirrhosis related osteoporosis. Ahmed et al. [4] suggested that leptin, a strong inhibitor of bone formation, may play a role in the pathogenesis of osteoporosis in postviral cirrhosis.

In our study, old age, female gender, low BMI, long duration of liver disease, and vitamin D deficiency have been found to be associated with low BMD, but none of them was an independent factor associated with bone disorders.

It is well established that the risk of osteoporosis increases with age, especially among females regardless of the presence of hepatic disease. The influence of these two factors on BMD in chronic liver disease was evaluated in several studies. One study [19] has found that the advanced age of patients was an independent risk factor of osteoporosis in patients with chronic liver disease. Female gender emerged in several series [3, 16] as a predictive factor of the occurrence of metabolic bone disorders particularly osteoporosis in patients with cirrhosis. Our study showed that patients with low BMD have a lower BMI compared with those with normal BMD. Our result was in agreement with Ormarsdóttir et al.'s finding [19]. In contrast, other studies [12, 20] did not find any correlation between BMI and osteoporosis or osteopenia. According to the authors, this finding can be biased by the overestimation of the weight of Child B or C patients due to the presence of ascites.

Javed et al. [13] showed that osteoporosis was more frequent in cirrhotic patients with long duration of liver disease (5 years and above) which is in agreement with our result.

The severity of cirrhosis assessed by the Child-Pugh score emerged in the majority of series [3, 4, 12, 14, 15] as being a factor which is strongly associated with a bone mass deficit especially osteoporosis. In our study, the Child-Pugh score did not influence BMD, and this can be explained by the fact that almost all patients in our study had a compensated cirrhosis (Child A), while few of them were Child B, unlike other studies in which the scores B and C of Child-Pugh classification were predominant. Our result agrees with Javed et al. [13] and Loria's finding; they showed no correlation between the severity of cirrhosis and BMD. The etiology of cirrhosis had no influence on BMD in our series, which agrees with the results of Javed et al. [13].

Most studies have not shown correlation between serum 25 (OH) D and the presence or severity of osteoporosis [21, 22]. In opposition to this, a high prevalence of vitamin D deficiency (95.6%) was found in our study, and it is associated with low BMD.

In hepatocellular dysfunction, some authors reported high levels of serum PTH [23], while others reported unchanged levels [3] or low level [12]. Bai et al. [24] showed that an increased level of PTH was an independent risk factor associated with low BMD. In our study, 28% of patients had a high PTH level with no difference between patients with normal BMD and those with osteoporosis or osteopenia.

Fractures occurrence is the most feared complication of hepatic osteodystrophy; a rate of 5 to 20% was reported in the literature [5]. Despite the high prevalence of metabolic bone disorders and vitamin D deficiency observed in our study, no case of vertebral or peripheral fracture was noticed during the study period.

This fracture risk in a patient's population already weakened by cirrhosis would justify a systematic evaluation of BMD in all cirrhotic patients in order to prevent this risk through the implementation of therapeutic measures appropriate to the degree of bone demineralization. The British [5] and American [25] societies of gastroenterology recommend actually BMD assessment in all patients with cirrhosis.

## 5. Conclusion

Our prospective study showed a high prevalence of BMD disorders in patients with viral cirrhosis with an osteoporosis rate of 28.3%. Old age, female gender, low BMI, long duration of liver disease, and vitamin D deficiency were found to be significantly associated with low BMD, but none of them was an independent factor associated with bone disorders. Consequently, BMD assessment and vitamin D dosage must be a part of systematic monitoring of viral cirrhosis in order to select patients with high risk of fractures requiring appropriate treatment.

## Figures and Tables

**Table 1:** Biochemical data of the 46 patients with viral cirrhosis.

Biological parameters	Values
ALT (UI/L)	58,38 [29–73,5]
AST (UI/L)	58,38 [29–73,5]
GGT (UI/L)	39 [34–114,5]
ALP (UI/L)	112,3 ± 46,54
Total bilirubin (mg/L)	8 [6,9–10,4]
Serum albumin (g/L)	38 ± 4,74
Prothrombin time (%)	80 ± 15
Serum calcium (mg/L)	91,1 ± 4,95
Serum phosphorus (mg/L)	33,14 ± 5,74
Urinary calcium (mg/24 h)	112,5 [63,2–197]
Urinary phosphorus (mg/24 h)	545,4 ± 255,8
25 (OH) D (*μ*g/L)	13,2 ± 5,1
iPTH (pg/mL)	55,4 [47–102]

BMI: body mass index; AST: aspartate aminotransferase; ALT: alanine aminotransferase; GGT: gamma-glutamyl transferase; ALP: alkaline phosphatase; 25 (OH) D: 25 hydroxy-vitamin D; iPTH: intact parathyroid hormone.

Variables are expressed as mean ± standard deviation or median (quartiles).

**Table 2:** Bone mineral density (g/cm^2^), *T*-score, and *Z*-score of all patients at the different measurement sites.

Measurement site	BMD	*T*-score	*Z*-score
Lumbar spine	0,98 ± 0,2	−1,59 ± 1,4	−0,64 ± 1,19
Left femur	0,88 ± 0,17	−0,98 ± 1,25	0,10 ± 1,03
Right femur	0,89 ± 1,62	−0,98 ± 1,19	0,05 ± 0,92
Total hip	0,88 ± 1,17	−0,99 ± 1,26	0,07 ± 0,99

BMD: bone mineral density. Data are expressed as mean ± standard deviation.

**Table 3:** Bone mineral density characteristics of the 46 included patients according to gender.

Gender	Men *n* = 26	Women *n* = 20	*P*
Lumbar spine			
BMD (g/cm^2^)	1,05 ± 0,18	0,89 ± 0,18	0,007
*T*-score	−1,19 ± 1,36	−2,12 ± 1,30	0,02
Total hip			
BMD (g/cm^2^)	0,94 ± 0,17	0,81 ± 0,14	0,01
*T*-score	−0,68 ± 1,27	−1,39 ± 1,16	0,04

BMD: bone mineral density. Variables are expressed as mean ± standard deviation.

**Table 4:** Clinical and biological characteristics of patients with normal BMD and those with low BMD.

Criteria	Normal BMD *n* = 9	Low BMD *n* = 37	*P*
Age (years)	57.8 ± 9.2	65.5 ± 8.4	0.02
Weight (kg)	71.3 ± 13.6	64.3 ± 8.7	NS
BMI (kg/m^2^)	26.7 ± 4.2	22.4 ± 1.8	0.01
Liver disease duration	6.6 ± 4.4	10.8 ± 5.3	0.04
Etiology (*n*)	VHC: 7/VHB: 2	VHC: 33/VHB: 4	NS
Child-Pugh score (*n*)	A: 7/B: 2	A: 33/B: 4	NS
Serum bilirubin (mg/L)	8 [6–11]	8 [6–9.6]	NS
Alkaline phosphatase (UI/L)	101 ± 34	114.5 ± 48.8	NS
Serum calcium (mg/L)	91.5 ± 4.6	89.3 ± 6.02	NS
Serum phosphorus(mg/L)	34.2 ± 4.8	32.8 ± 5.9	NS
24 h urinary calcium (mg/24 h)	175.2 [117–245]	86 [58.2–181.4]	NS
24 h urinary phosphorus (mg/24 h)	774.3 ± 249.9	550 ± 224.1	NS
25 hydroxy-vitamin D (*μ*g/L)	16.8 ± 3.9	12.3 ± 5	0.01
Parathyroid hormon (pg/mL)	46.4 [24–116]	59 [47–102]	NS

BMD: bone mineral density; BMI: body mass index; VHB: viral hepatitis B; VHC: viral hepatitis C; NS: nonsignificant. Data are expressed as mean ± standard deviation or median (quartiles).

**Table 5:** Comparative statistical study of clinical and biological characteristics of patients with metabolic bone disorders and patients with normal BMD.

	Univariate logistic regression			Multivariate logistic regression		
	OR	CI	*P*	OR	CI	*P*
Age	1,09	[1,01–1,19]	0,03	1,06	[0,80–1,41]	0,67
Weight	0,93	[0,87–1,01]	0,07			
BMI	0,55	[0,37–0,84]	0,006	0,70	[0,38–1,29]	0,26
Duration of liver disease	1,18	[1,02–1,39]	0,04	1,33	[0,88–2,01]	0,16
25 Hydroxy-vitamin D	0,82	[0,69–0,97]	0,02	1,02	[0,72–1,45]	0,90
Parathyroid hormone	1,01	[0,97–1,04]	0,68			

OR: odds ratio; CI: confidence interval; BMD: bone mineral density.
